# Prevalence of Kawasaki Disease in a Tertiary Care Hospital: A Descriptive Cross-sectional Study

**DOI:** 10.31729/jnma.4746

**Published:** 2019-12-31

**Authors:** Kripa Rajak, Anu Radha Twayana, Rashmi Shrestha, Puja Amatya, Calvin Ghimire

**Affiliations:** 1 Patan Academy of Health Sciences, Patan, Nepal; 2 Department of Oxford University Clinical Research Unit, Patan Academy of Health Sciences, Patan, Nepal; 3 Department of Paediatrics, Patan Academy of Health Sciences, Patan, Nepal

**Keywords:** *Kawasaki disease*, *prevalence*, *vasculitis*

## Abstract

**Introduction:**

Kawasaki disease is an acute vasculitis of unknown etiology. The epidemiological data available for Nepal remains insufficient. In Nepal, Kawasaki disease has only been reported in cases of brief reports, leaving the true disease burden unknown. Many cases go undiagnosed and untreated due to a lack of knowledge regarding this entity. The objective of this study was to find the prevalence of Kawasaki disease in a tertiary care hospital.

**Methods:**

This descriptive cross-sectional study was carried out in a tertiary care hospital of Nepal from 2013 to 2018 after taking ethical approval from the Institutional Review Committee. The sample size was calculated and the consecutive sampling method was done. Data collection and entry was done in Microsoft Excel, point estimate at 99% Confidence Interval was calculated along with frequency and proportion for binary data.

**Results:**

The overall prevalence of Kawasaki disease was found to be 0.10% at 95% Confidence Interval (0.07-0.13%) among 11,416 patients under the age of 5 years admitted in pediatrics ward. There were 4 (33.33%) cases of complete Kawasaki and 8 (66.67%) cases of incomplete Kawasaki. There were 9 (75%) males and 3 (25%) females and the male to female ratio was 3:1. There was a male preponderance. The age at diagnosis ranged between 4 and 60 months. The median age at diagnosis was 10.5 months. The most common presentation was fever, conjunctivitis, rash, and oral changes.

**Conclusions:**

Prevalence of Kawasaki disease was found to be lesser compared to other studies done in other countries. Knowledge of Kawasaki disease among Nepalese pediatricians should be enhanced to guarantee the appropriate diagnosis and treatment of this disease.

## INTRODUCTION

Kawasaki disease (KD) is a systemic vasculitis that mainly affects children younger than 5 years.^1^ First described in Japan in l967 by Tomisaku Kawasaki,^2^ currently, KD has been diagnosed in Asia, the Middle East, Latin America, and Africa, as well as in North America and Europe.^1^ KD is characterized by fever, bilateral non-exudative conjunctivitis, erythema of the lips and oral mucosa, changes in the extremities, rash, and cervical lymphadenopathy.^2^ Coronary artery aneurysms or ectasia develop in approximately 15% to 25% of untreated children and may lead to ischemic heart disease or sudden death.^2^

It is usually under-diagnosed due to lack of knowledge regarding this entity among pediatricians and dermatologists.^3^ In Nepal, KD has only been reported in cases of brief reports, leaving the true disease burden unknown. Many cases go undiagnosed and untreated due to the lack of knowledge regarding this entity.

This study aims to find the prevalence of KD in a tertiary care hospital and to find the most common clinical presentation among these patients.

## METHODS

This descriptive cross-sectional study was conducted at Patan Academy of Health Sciences, Patan, Nepal from 2013 to 2018. Following approval from Institutional Review Committee, all patients under the age of 5 years admitted in the paediatric ward of Patan Academy of Health Sciences were included in the study. Among them, patients with the discharge diagnosis of KD were identified based on the International Classification of Diseases (ICD) code for KD by the software used in the record section of the hospital.^2^ These data were crossreferenced with those from the audit of the Department of Paediatrics. The sample size was calculated as mentioned below:

$$\begin{array}{ll} \text{n} & ={\text{Z}}^{\text{2}}\times \text{(p}\times \text{q)}/{\text{e}}^{\text{2}}\\ & ={(\text{2.6)}}^{\text{2}}\times (\text{0.5}\times \text{0.5)}/{(\text{0.02)}}^{\text{2}}\\ & =4225 \end{array}$$

Where,

n= required sample size
Z= 2.6 for 99% Confidence Interval
p= prevalence of Kawasaki Disease (50%)
q= i-p
e= margin of error, 2%

The minimum sample size was calculated to be 4225. Since this study used a non-randomized sampling method (consecutive sampling), we have doubled the sample. Thus the calculated sample size was 8850. We have taken a sample of 11,416. Demographic data was taken from the hospital record.

Reporting bias were minimized as possible. Data were entered and calculations were done using Microsoft Excel, point estimate at 99% CI was calculated along with frequency and proportion for binary data.

## RESULTS

The overall prevalence of Kawasaki Disease was found to be 12 (0.1%) among 11,416 patients under the age of 5 years admitted in paediatrics ward over a period of six years. There were 4 (33.33%) cases of complete Kawasaki and 8 (66.67%) cases of incomplete Kawasaki.

**Figure 1:**
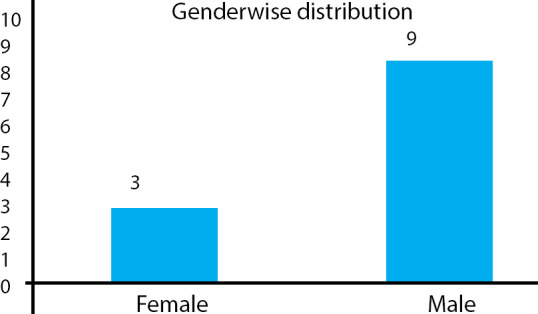
Gender distribution of patients diagnosed with KD.

There were 9 (75%) males and 3 (25%) females and the male to female ratio was 3:1. There was a male preponderance. Provisional diagnoses made at the time of admission in most of the cases were scarlet fever, urinary tract infection, occult bacterial infection, and measles (Table 1). KD was common among males 9 (75%) compared to females 3 (25%) (Figure 1).

**Table 1:** Clinical presentation of patients at diagnosis.

Diagnosis criteria	n (%)
Fever lasting at least five days	12 (100)
Changes in extremities	6 (50)
Oral mucosa changes	7 (58)
Rash	7 (58)
Conjunctivitis	7 (58)
Cervical lymph node	5 (42)

**Figure 2:**
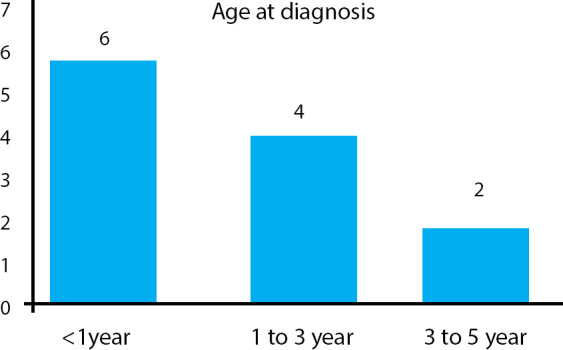
Age distribution of patients with KD.

The age at diagnosis ranged between 4 and 60 months. The median age at diagnosis is 10.5 months (Figure 2).

**Figure 3:**
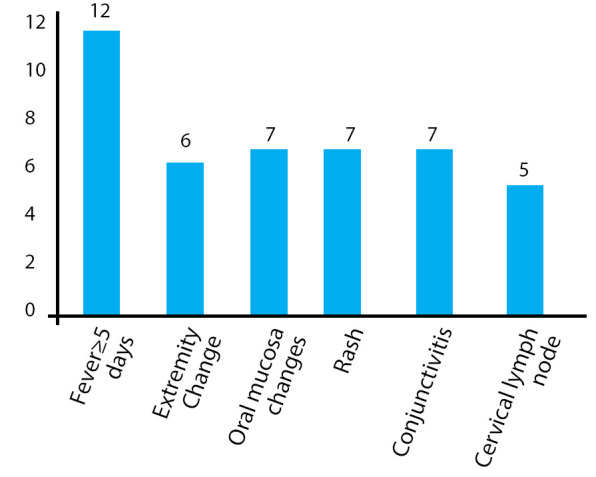
Clinical features in Kawasaki disease.

The mean duration of fever was 10±2 days and the range of temperature was 102±2°F (mean 102.5°F). Non-diagnostic clinical features reported were respiratory symptoms like cough which was the commonest 7 (58%) followed by gastrointestinal 7 (58%) symptoms like decreased feeding, vomiting, abdominal pain, diarrhoea and genitourinary 2 (16%) like sterile pyuria. Two of the patients (16%) reported irritability as part of the acute presentation (Figure 3).

Leukocytosis was seen in all cases 12 (100%). Similarly, 12 (100%) cases had increased sedimentation rate (≥20mm/h) and C-reactive protein (CRP) (≥10mg/l). Anaemia, hypoalbuminemia, elevated liver enzymes and sterile pyuria were the other laboratory findings (Table 2).

**Table 2:** Laboratory parameters of patients with KD.

Variables	Mean Value±SD	Range
Leucocyte (×10^3^/mm^3^)	24.53±6.64	8.2-31.4
Platelets (×10^3^/mm^3^)	542.4±261.23	220-1207
ESR (mm/1^st^ hour)	102±32.66	40-133
CRP (mg/L)	196.8±223.37	65-818

Of the 12 patients, only 1 (8%) case had evidence of cardiac complication i.e. coronary artery abnormality.

## DISCUSSION

The prevalence of KD was 0.1% at Patan Academy of Health Sciences. KD mainly occurred in male patients and in <5 years of age similar to previous reports from other countries.^4^ The most common presenting symptoms were fever, conjunctivitis, rash, oral and extremity changes. The male to female ratio was 3:1. There seemed to be delay in the diagnosis of the disease as the mean duration of the fever was long (10±2 days) and only a few cases were diagnosed within the six years study period. Most delays are due to difficulties in distinguishing KD from bacterial and viral infections, lack of awareness of KD among practicing pediatricians, general practitioners, and residents or incomplete presentation of clinical symptoms.^5-7^ No study reporting incidence of KD has been published from Nepal. As of now, detailed information about this disease is not available in Nepal.

In our study, we analyzed the frequency of clinical signs. The most common symptom was fever noted in all patients which were similar to the findings reported by Yadav, et al.^3^ In our study, of the five cardinal symptoms; extremity changes, mucosal changes, and polymorphous rash occurred most frequently. This was not consistent with the results reported by other authors which showed conjunctival injection, mucosal changes, and rash as the most frequent symptoms.^3,7^ Our study showed conjunctival findings in 58% cases which were less as compared to other studies. The reasons for this may be due to late presentation or reporting of cases, parents unaware of the eye changes or atypical presentations. Other non-cardinal symptoms of KD have been reported in the literature. Our study found nondiagnostic clinical features like cough, abdominal pain, decreased feeding, vomiting, diarrhea, irritability, sterile pyuria which were similar to what other studies had reported.^3,7^ Unlike other studies,^3,6,7^ our study showed one case of coronary abnormalities. The reason behind the cardiac complication in only one patient might be due to under-diagnosis of the complications due to lack of follow up or due to the diagnosed cases had received immunoglobulin and aspirin timely. This result might suggest the positive effect of immunoglobulin in treating KD and preventing coronary artery abnormalities as there are studies suggesting an increased risk of developing CAA due to delayed administration of IVIG.^8^

As the data collection was done from the hospital records, some cases of KD were missing. KD is a rare disease and in addition, we included only the cases which were admitted in the Paediatrics ward. This might have caused the cases who refused admission to be missed.

This was a single center-based descriptive crosssectional study done from the hospital record of admitted patients. So, patients who presented to OPD with the suspicious diagnosis of KD and refused an investigation, as well as admission, were missed and thus actual number could be under-reported. Generalizability is limited so our findings become rather suggestive.

## CONCLUSIONS

These results suggest that cases of Kawasaki disease are prevalent in Nepal and it needs to be further studied in multiple centers. Knowledge of Kawasaki disease among Nepalese pediatricians should be enhanced to guarantee the appropriate diagnosis and treatment of this disease.

## Conflict of Interest

**None.**
